# A CMOS-Compatible, Low-Noise ISFET Based on High Efficiency Ion-Modulated Lateral-Bipolar Conduction

**DOI:** 10.3390/s91008336

**Published:** 2009-10-21

**Authors:** Sheng-Ren Chang, Hsin Chen

**Affiliations:** Institute of Electronics Engineering, National Tsing Hua University, 30013 HsinChu, Taiwan; E-Mail: d935040@oz.nthu.edu.tw (S.-R.C)

**Keywords:** ISFET, low noise, CMOS-compatible, lateral-bipolar conduction

## Abstract

Ion-sensitive, field-effect transistors (ISFET) have been useful biosensors in many applications. However, the signal-to-noise ratio of the ISFET is limited by its intrinsic, low-frequency noise. This paper presents an ISFET capable of utilizing lateral-bipolar conduction to reduce low-frequency noise. With a particular layout design, the conduction efficiency is further enhanced. Moreover, the ISFET is compatible with the standard CMOS technology. All materials above the gate-oxide are removed by simple, die-level post-CMOS process, allowing ions to modulate the lateral-bipolar current directly. By varying the gate-to-bulk voltage, the operation mode of the ISFET is controlled effectively, so is the noise performance measured and compared. Finally, the biasing conditions preferable for different low-noise applications are identified. Under the identified biasing condition, the signal-to-noise ratio of the ISFET as a pH sensor is proved to be improved by more than five times.

## 1. Introduction

Field-effect transistors have been employed as biosensors to detect ion concentration, biomolecules, neural activity, etc. [1-6]. In these applications, a large sensor array is becoming essential for detecting multiple biomolecules or for interfacing multiple biological cells in parallel [7-10]. This demand leads to at least two challenges. First, integrating the sensors with signal-processing circuits on a single chip is important to reduce wiring complexity and noise interferences. Second, the low-frequency noise of field-effect sensors has to be further reduced for recording weak biomedical signals such as neural activity, which could be less than tens of micro-volts in magnitude.

A variety of methods has been proposed to integrate field-effect sensors with the standard CMOS technology [11-13], the prominent technology for fabricating integrated circuits. However, micromachining processes become limited and only applicable after the CMOS process in a constrained condition. To avoid complex post-CMOS processing, most CMOS-compatible, field-effect sensors simply employ the passivation layer (silicon nitride/silicon oxynitride) as the surface material, and using a floating gate formed by metals to couple the potential changes at the sensory surface [13-15]. Compared to the first ISFET with gate replaced by an aqueous solution [16], the floating-gate ISFET requires a larger sensory area (several hundreds of μm^2^) to ensure sufficient sensitivity. However, applications like neural recording desire a pitch size smaller than a single neuron (around 20 μm) [6,17,18]. ISFETs with the discrete-gate structure [19,20], or the open-gate structure [21,22], have thus been proposed. However, the open-gate structure in [22] is created by plasma etching, which could damage the ISFET easily or introduce extra mismatches [23].

**Figure 1.**
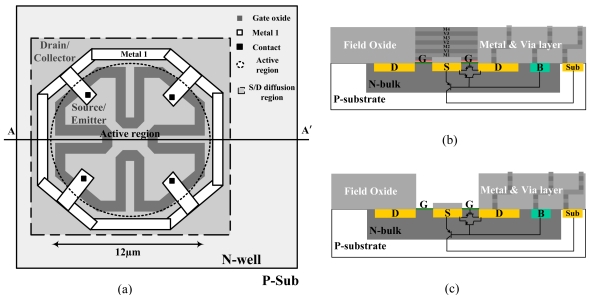
(a) The layout of the proposed ISFET (b) The cross-sectional view before the post-CMOS process (c) The cross-sectional view after the post-CMOS process.

As most biomedical signals have a frequency bandwidth below 10 kHz [24], the low-frequency noise of a field-effect transistor dominates to limit the signal-to-noise ratio of recording. One simple approach is increasing the transistor size [25,26], but this again limits the minimum pitch size achievable. As low-frequency noise is closely related to charge trapping at the oxide-silicon interface, the study in [27] demonstrates that forward-biasing the source-to-bulk junction also helps to reduce low-frequency noise. While such noise reduction could not be well explained by the models of the flicker noise [27,28], one possible explanation is that the forward-biasing encourages the lateral-bipolar conduction, avoiding interface traps and thus reducing noise [29,30]. However, the main drawback of the lateral-bipolar conduction is the leakage current through the parasitic, vertical bipolar transistor (Figure 1).

Based on the post-CMOS process proposed in [23], this paper presents a CMOS-compatible ISFET able to operate both in the metal-oxide-semiconductor (MOS) mode and in the lateral-bipolar junction transistor (LBJT) mode. The LBJT conduction allows noise to be reduced significantly for low-noise applications. The layout technique is further applied to define a particular structure, reducing the leakage current of the bipolar conduction. In addition, all materials above the gate-oxide of the ISFET are removed by the die-level, post-CMOS process, allowing the ions to modulate the drain current directly, so as to enhance the sensitivity. Following the introduction, Section 2 describes the design, fabrication, and the measurement setup of the ISFET. All the measurement results are presented and discussed in Section 3. Finally, Section 4 concludes the findings and points out future works.

## 2. Experimental Section

### 2.1. The layout and the structure of the ISFET

Figure 1(a) shows the layout of the ISFET for fabrication with the TSMC 0.35 μm CMOS technology. As the chip is returned from the foundry, the cross-sectional view along the line AA′ is shown in Figure 1(b). The cross-sectional view then becomes Figure 1(c) after the post-CMOS process. In Figure 1(a), the dark-grey, continuous line segments represent the polygate mask, defining the channel region of the transistor. The dashed-dot rectangle enclosing the polygate then defines the highly-doped, p-type diffusion region. The region enclosed by the polygate thus corresponds to the source terminal. A metal line is added to interconnect the source diffusion at different corners, reducing the parasitic resistance. The diffusion region outside the polygate corresponds to the drain terminal. The dashed circle in Figure 1(a) indicates the active region of the sensor, within which all materials above the gate-oxide is removed by the post-CMOS process. As shown by Figure 1(b), the active region is defined by stacks of metal layers. The passivation above the top metal layer is already removed as the chip is returned from the foundry, allowing all metals and the polygate (denoted as G) to be removed by wet-etching.

Figure 1(c) reveals the post-processed ISFET with its parasitic transistors. Let the drain voltage (V_D_) be constant and lower than the voltages of all other terminals. With different gate-to-bulk voltages (V_GB_) and source-to-bulk voltages (V_SB_), the ISFET can operate in the MOS mode, or the LBJT mode [29], or the hybrid of both modes [31]. In the MOS mode, both V_GB_ and V_SB_ are negative. As V_GS_ is smaller than the threshold voltage (V_TP_), a channel (inversion layer) is induced at the oxide-silicon interface. A positive V_SD_ then causes the current to flow along the channel, experiencing low-frequency noise relating to interface traps. Even if V_TP_ < V_GS_ < 0, the subthreshold current still conducts along the oxide-silicon interface by the diffusion process, resulting in an even worse signal-to-noise ratio. In the LBJT mode, both V_GB_ and V_SB_ are positive. The forward-biased source-bulk junction induces injection current that conducts through the LBJT formed by the source, bulk, and drain regions, corresponding to the emitter, base and collector terminals, respectively. Together with a large, positive V_GB_, the current is repelled away from the oxide-silicon interface, so is the noise reduced [29]. If the V_GB_ is small or even negative such that V_GS_ < 0 and V_SB_ > 0, the ISFET operates in the hybrid mode. The current conducts through both the MOS and the LBJT transistors. The proportion of the current in each transistor is modulated by V_GB_, so is the noise.

The main drawback of the LBJT conduction is the unavoidable leakage current through the vertical bipolar junction transistor (VBJT), which is always activated together with the LBJT. The leakage current not only introduces extra power consumption but also puts the chip in the risk of latch-up. In response to this drawback, our design has the source region completely surrounded by the drain region (Figure 1(a)), enhancing the collection of hole currents for the LBJT. In addition, the polygonal structure of the gate maximizes the W/L ratio (63 μm/0.96 μm), i.e., the emitter-base junction area of the LBJT, within the finite active region. It is notable that the minimum channel length is not used in order to ease the poly-gate etching.

### 2.2. The post-CMOS process

**Figure 2.**
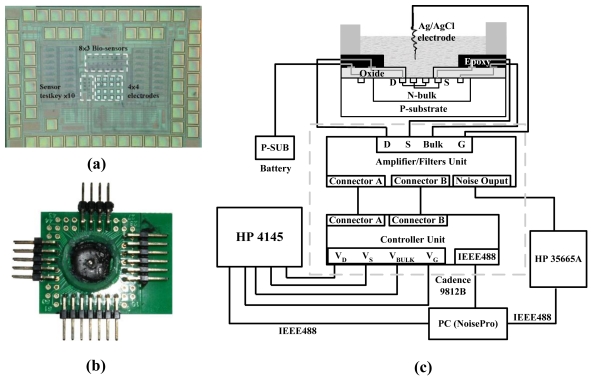
(a) The chip containing the testkeys of the proposed ISFET; (b) The packaged chip after post-CMOS process; (c) The schematic of the noise measurement system.

The die-level, post-CMOS process for removing the materials in active region had been detailed and carefully verified in [23]. The testkeys of the proposed ISFET were included in the chip shown in Figure 2(a). The chip also contained multi-finger ISFET arrays integrated with recording amplifiers and multiplexers, whose functionality had been tested and reported in [23,35]. After the chip was fabricated with the standard CMOS process, the metal layers were first removed by wet etching with “piranha” solution (H_2_SO_4_:H_2_O = 2:1) at 85 °C for around 80 s until the polygate was exposed. The thin silicide layer above the poly-gate was then removed by the reactive-ion etching (RIE) for five minutes. Afterwards, the polygate was removed by wet etching with diluted KOH (KOH: DI water = 2:1 by weight) at 80 °C for around 20 seconds. With a shadow mask formed by a fragment of a silicon wafer, the passivation layer above the bonding pads was removed by the RIE. The chip was then wire-bonded to a printed circuit board, and the bonding wires were coated with the industrial epoxy (WK-8126H, WinKing). Finally, a glass O-ring was attached to the PCB to form a tank for containing solutions above the chip, as shown in Figure 2(b).

### 2.3. Noise Measurement

Figure 2(c) shows the measurement setup including a semiconductor parameter analyzer (HP 4145), a dynamic signal analyzer (HP 35665A), and a noise analyzer (Cadence 9812B). The parameter analyzer generated direct-current (DC) biases for the ISFET, and measuring the corresponding current or voltage responses. Under different biasing conditions, the noise was first amplified by the noise analyzer, and then measured by the dynamic signal analyzer. All the equipments were configured by the software “NoisePro”.

### 2.4. pH Sensing

**Figure 3.**
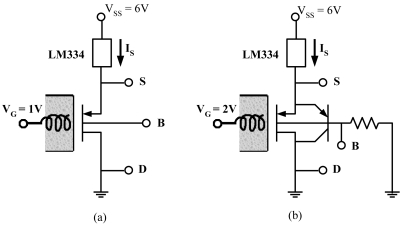
The biasing circuits for the ISFET to function as a pH sensor. (a) The ISFET operates in MOS mode (b) The ISFET operates in the LBJT or the hybrid mode.

To function as a pH sensor, the ISFET was biased with a constant drain current and thus a constant *V_GS_*. As the effective threshold voltage (V_TP_) changed with hydrogen concentrations, the source voltage (*V_S_*) simply followed the changes of V_TP_. Figure 3 illustrated the schematics of the biasing circuits. In the MOS mode (Figure 3(a)), *V_G_* = 1 V, *V_B_* = 1.5 V, and a current source (Nat. Semi. LM334) at the source terminal forced the ISFET to conduct a constant current. In the LBJT mode or the hybrid mode (Figure 3(b)), *V_G_* = 2 V and a resistor of 2 MΩ is connected between the bulk and the ground. The resistance was selected to bias *V_B_* around 1.5 V. The large resistance caused the base current and thus the emitter current of the LBJT to be nearly constant. Together with the current source at the source terminal, the current flowing through the MOS transistor was also kept constant. As a result, *V_GS_* and *V_SB_* were fixed, allowing *V_S_* to follow the change of the effective threshold voltage. With the ISFET biased in different modes, the responses of *V_S_* to the pH-values of solutions were measured and compared.

## 3. Results and Discussion

### 3.1. Device fabrication

**Figure 4.**
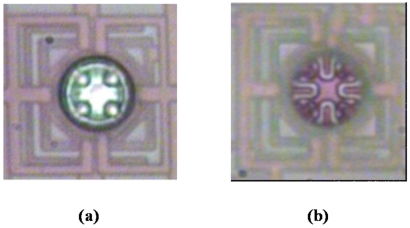
(a) Sensor without post-CMOS process (b) Sensor after post-CMOS process.

Figure 4(a) shows a microphotograph of the ISFET, taken when the chip was just returned from the foundry. The shining of the metals within the active region was clearly visible. After the post-CMOS process, the shining disappeared completely, as shown in Figure 4(b), indicating the complete removal of metal layers. The white line segments within the circle corresponded to the gate oxide of the ISFET, and the light-red regions to the source diffusion covered by a thicker oxide. Although observing the colors was not sufficient for confirming the complete removal of poly-gates, the duration of KOH-etching has been optimized according to [23]. After the chip was packaged as Figure 2(b), physiological buffer (210 mM NaCl; 15 mM CaCl2, 5.4 mM KCl, 2.6 mM MgCl_2_, and 5mM HEPES, pH 7.4) was filled in the glass O-ring, and an Ag/AgCl wire was immersed in the buffer to define the solution potential, i.e., the effective gate voltage of the ISFET.

### 3.2. Operation modes and efficiency

**Figure 5.**
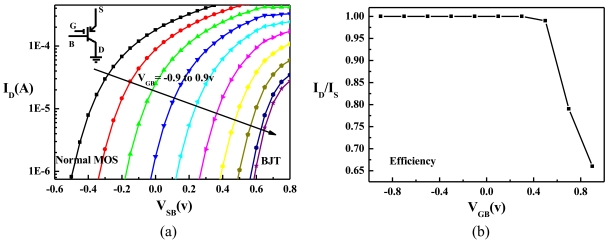
(a) The measured *I_D_-V_SB_* characteristics of the ISFET for various *V_GB_*. (b) The measured efficiency (*I_D_/I_S_*) against *V_GB_* of the ISFET when *I_S_* is fixed at 10 μA. Operation modes are changed from MOS mode to the LBJT mode by increasing the gate voltage.

As the source voltage (*V_S_*) was swept from 1.0 V to 2.3 V with the solution potential (*V_G_*) stepping from 0 V to 2.5 V with a stepsize of 25 mV, the drain current (*I_D_*) and the source current (*I_S_*) of the ISFET were measured. The bulk and the drain voltages were kept constant (*V_B_* = 1.5 V, *V _D_* = 0 V) during the measurement. Figure 5(a) plots *I_D_* against *V_SB_* for various *V_GB_*. The current-voltage relationship was analogous to that measured from an ordinary LBJT with its gate remaining [29], indicating that the gate-oxide of the ISFET had been successfully preserved from the post-CMOS process. Moreover, Figure 5(a) revealed that the operation mode of the ISFET shifted from the MOS mode to the LBJT mode as *V_GB_* increased from −0.9 V to 0.9 V. After *V_GB_* > 0.9 V, the *I_D_-V_SB_* curves overlapped with the rightmost curve in Figure 5(a) because *I_D_* was entirely conducted through the LBJT. Therefore, ions in the solution can only modulate *I_D_* effectively when *V_GB_* < 0.9 V, i.e., when the ISFET operated in the MOS mode or the hybrid MOS-LBJT mode (*V_SB_* > 0).

Let the efficiency of the ISFET be defined as the ratio between *I_D_* and *I_S_*. The efficiency is high when the leakage current through the VBJT is small. Figure 5(b) plots the efficiency against *V_GB_* when *I_S_* is fixed to be 10 μA. The efficiency clearly depends on *V_GB_*, which determines the portion of *I_D_* conducting through the LBJT. The efficiency is greater than 95% as *V_GB_* ≤ 0.5 V, and decreasing quickly to 65% as *V_GB_* = 0.9 V. This indicates that LBJT conduction starts to dominate after *V_GB_* > 0.5 V, so is the leakage current increased significantly. Nevertheless, by the merits of our layout design, the efficiency remains more than 90% when 0 V < *V_GB_* < 0.6 V, within this range, the ISFET operates in the hybrid mode, allowing ions to modulate *I_D_* with not only reduced noise but also high efficiency.

### 3.3. Transconductance

**Figure 6.**
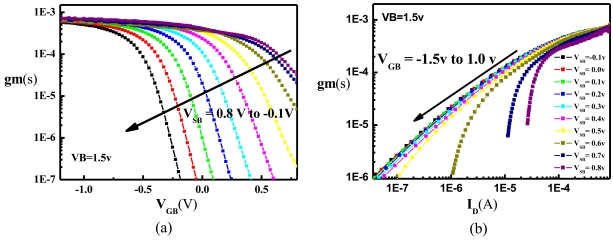
The measured (a) *g_m_-V_GB_* (b) *g_m_-I_D_* of the ISFET for various *V_SB_*.

The transconductance (*g_m_*) of the ISFET is defined as the derivative of *I_D_* with respect to *V_GB_*. A larger *g_m_* provides a better sensitivity to the potential changes within the solution. Figure 6(a) plots *g_m_* against *V_GB_* with various *V_SB_*, revealing that *g_m_* decreases with increasing *V_GB_*. This is because positive *V_GB_* repels the current away from the oxide-silicon interface, reducing the modulating capability of the solution potential. With a specific *V_GB_*, the *g_m_* also decreases with increasing *V_SB_* because increasing *V_SB_* forward-biases the source-bulk junction, causing more currents to flow through the LBJT. Figure 6(b) further plots *g_m_* against *I_D_* for various *V_SB_*, showing clearly that a larger *I_D_* is required for achieving the same *g_m_* as more current is conducted through the LBJT. Therefore, a compromise exists among the noise, the efficiency, and the transconductance. If more current conducts through the LBJT, the noise is smaller but the efficiency and the transconductance are degraded. The optimum biasing point for the ISFET is thus application-dependent, as discussed in Sections 3.4 and 3.5.

### 3.4. Noise performance with constant I_D_

As the ISFET is employed to detect ion concentration or biomolecules, the ISFET is normally biased with a constant *I_D_* [32,33]. When ions or biomolecules change the effective threshold voltages, the corresponding changes at *V_S_* are recorded. Since the voltage signal is recorded directly, the transconductance of the ISFET is not of concern. The noise can thus be reduced by increasing *V_GB_* to repel current conduction away from the oxide-silicon interface.

**Figure 7.**
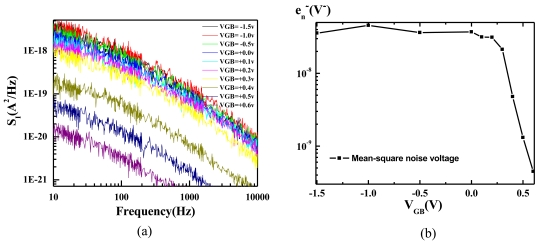
Noise measured with various V_GB_ as the ISFET was biased with a constant *I_D_*. (a) noise power spectral density (b) mean-square noise voltage integrated from 10 to 10 kHz

Figure 7(a) shows the noise spectrum measured as the ISFET is biased with *I_D_* = 10 μA and various *V_GB_*. The noise decreases by several decades as *V_GB_* increases from −1.5 V to 0.6 V. By integrating the noise spectral over the frequency range of most biomedical signals (10–10 kHz) and dividing the integrated value by *g_m_^2^*, the total mean-square noise voltage (*e_n_^2^*) referred to the sensory surface is obtained and plotted in Figure 7(b). The noise reduces significantly after *V_GB_* > 0 V. Although the noise continues to reduce with increasing *V_GB_*, the efficiency of the ISFET degrades after *V_GB_* > 0.5 V (Figure 5(b)). Therefore, *V_GB_* = 0.5 V is a biasing point that achieves a good compromise between efficiency and noise for the proposed ISFET.

### 3.5. Noise performance with constant g_m_

**Figure 8.**
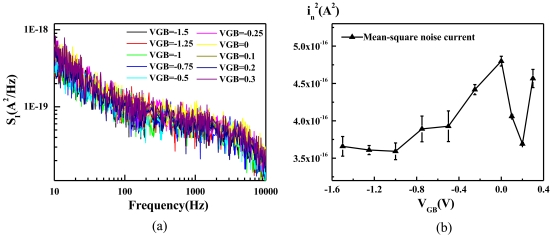
Noise measured with various V_GB_ as the ISFET was biased with a constant *g_m_*. (a) noise power spectral density (b) mean-square noise current integrated from 10 to 10 kHz.

As the ISFET is employed to detect transient potential changes (e.g., neural activity), the potential changes are normally transduced into drain currents for further amplification and filtering [34,35]. A large transconductance (*g_m_*) is thus important for ensuring the sensitivity of the ISFET. As both noise and *g_m_* decrease with *V_GB_*, an optimum *V_GB_* could exist for the hybrid-mode operation (0 V < *V_GB_* < 0.6 V). Therefore, by biasing the ISFET with a constant *g_m_* = 0.1 mS, the noise spectral density for various *V_GB_* is measured and shown in Figure 8(a).

The total mean-square noise current (*i_n_^2^*) integrated from 10 to 10 kHz is shown in Figure 8(b), where the error bars represent the standard errors across two devices. For *V_GB_* < −0.8 V, MOS conduction in the saturation mode dominates. In this operating mode, the same *I_D_* gives the same *g_m_*. As the low-frequency noise is simply proportional to *I_D_* [28], the noise power is roughly the same for different *V_GB_*. As −0.8 V < *V_GB_* < 0 V, the MOS enters the substhreshold operation. Increasing *V_GB_* causes interface traps to be more negatively-charged, and thus easier to attract hole currents to induce noise [20]. Most important of all, as *V_GB_* > 0 V, the operation shifts into the hybrid mode. A minimum occurs at *V_GB_* = 0.2 V. As discussed in Section 3.3, the minimum comes from the fact that increasing V_GB_ encourages LBJT conduction to reduce noise while causing *g_m_* to reduce at the same time. A higher *V_GB_* thus requires a higher biasing current to maintain *g_m_* = 0.1 mS, but the noise increases with the biasing current. Therefore, the biasing point at *V_GB_* = 0.2 V achieves a good compromise between the transconductance and the noise. Beyond this point, the increase of noise with the biasing current becomes non-negligible. Although a local optimum point exists for the hybrid operation, the noise level in the hybrid mode is not significantly lower than that in the MOS mode. The only difference is that the noise in the MOS mode exhibits larger variation, owing to the fact that the noise is closely related to the interface traps, whose amount varies easily from one device to another. In addition, the noise reduction in the hybrid mode is much less significant than that achieved in the constant-*I_D_* case (Figure 7(b)). Therefore, to use the ISFET as a potentiometric sensor, biasing the ISFET as a source follower as shown in Figure 3(b) is still preferable.

### 3.6. pH sensing

**Figure 9.**
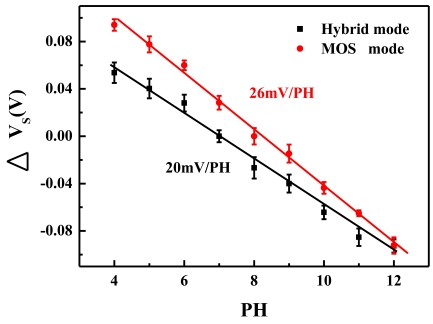
The measured responses of *V_S_* to solutions with different pH values as the ISFET was biased in the MOS mode (red) and in the hybrid mode (black).

Section 3.4 indicated that the ISFET with *V_GB_* = 0.5 achieved a good compromise between high efficiency and small noise. To verify the noise reduction improved the signal-to-noise ratio, the sensitivity of the ISFET operating in different modes was further measured and compared in the context of pH sensing. The red circles in Figure 9 showed the measured responses of *V_S_* as the ISFET was biased in the MOS mode with *V_GB_* = −0.5 V (Figure 3(a)). On the other hand, the black squares showed the responses of *V_S_* as the ISFET was biased in the hybrid mode with *V_GB_* = 0.5 V (Figure 3(b)). The error bars indicated the standard deviation over four measurements. By fitting the results with the red and black lines, the sensitivity was found to be 26 mV/pH for the MOS mode and 20 mV/pH for the hybrid mode. As the mean-square noise voltage with *V_GB_* = −0.5 was around 35 times larger than that with *V_GB_* = 0.5 (Figure 7(b)), it is clear that the hybrid mode improves the signal-to-noise ratio by around five times.

## 4. Conclusions

A CMOS-compatible ISFET capable of operating in both MOS and LBJT modes has been fabricated and tested. The special structure defined by layout masks is proved to facilitate operation in the hybrid MOS-LBJT mode with high efficiency and low noise. With constant-current biasing, the noise of the ISFET can be reduced by two decades by adjusting the gate-to-bulk voltage (*V_GB_*). As transconductance is of concern, an optimum *V_GB_* exists for the ISFET to have sufficient transconductance, high efficiency, and minimized noise. As the ISFET can be fabricated by simple post-CMOS process at the die level, the ISFET will be integrated with signal-processing circuits on a single chip for low-noise, biomedical applications.
